# Case of Rupture of the Uterus

**Published:** 1876-11

**Authors:** Isaac G. Porter

**Affiliations:** New London, Conn.


### CASE OF RUPTURE OF THE UTERUS;

### REMARKABLE FOR ITS COMPARATIVE CAUSELESSNESS, SO FAR AS KNOWN, AND THE ABSENCE OF THE USUAL ANTECEDENTS.

BY ISAAC G. PORTER, M.D., NEW LONDON, CONN.

*April* 10, 1875.—Called at 7 A.M. to Mrs. G., residing four miles distant; æt. thirty-nine years; mother of three children, the youngest born two years since. Having been with her at that time, and also at the preceding birth, I can testify that her labors were short, and in every respect favorable. There existed, however, a constitutional tendency to obesity and relaxation. Found, on arrival, that her pains, which had been slight, had subsided, and after waiting an hour, an examination was instituted to learn the condition and progress of the labor. It was with difficulty that the os uteri, which was closed, could be reached, so high was it in the vagina. Anterior obliquity was strongly marked. The day having passed without pain, I was again summoned in the evening, arriving between 8 and 9 o'clock. The labor was far from active; indeed, another false alarm was anticipated. The position of the uterus was as before described, the os being slightly dilated, and still reached with great difficulty, owing, as I thought, to extreme anterior obliquity. To remedy this, a finger was inserted within the os, and slight traction made to draw it forward, while lifting efforts with towels beneath the abdomen, externally, were carefully made, the patient lying on her back. At 10 o'clock the pains became somewhat more active, but were far from satisfactory, and so continued until 11:30 P.M., when, for a few minutes, they became strong, being aided somewhat by voluntary efforts, but not violent. So little benefit did I expect from them, that, to avoid unnecessary assistance and to abridge the tedium of waiting, I was sitting in an adjoining room, the connecting door being open, when the attendants came rushing in, saying that something had burst in her last pain, and that she was in great agony. I found her countenance greatly changed, hands and feet bathed in a cold sweat. The pains had ceased, of course, and there was slight flooding. A vaginal examination revealed a large rupture of the cervix, the hand readily reaching the head of the child, while the body was still covered by the uterus. A consulting physician, Dr. R. A. Manwaring, soon arrived, and it was thought advisable to deliver by turning, which I accomplished with little difficulty; the child, however, being dead. The placenta soon followed, and the patient rallied fairly, and subsequently gave some promise of recovery. She died, however, on the fourth day. The child, I may add, was not disproportionally large.

Two years before, as stated, after an easy labor, she was delivered of a full-grown child. Is it probable that, within the period named, any permanent obstruction could have formed to prevent the descent of the head at the superior strait? In Dr. Trask's series of cases, anterior obliquity is only once assigned as the cause of the accident. Doubtless, anything which impedes the course of labor exerts an influence in favor of rupture. In an entirely healthy uterus, the existing obliquity may not have been adequate to produce the result, but it is not so when conjoined, as it may have been in this case, with fatty degeneration, friability, atrophy, or thinning—causes, all of which are usually recognized in most treatise on this subject, and particularly by Drs. Murphy and Trask. It is to be regretted that, through distance and other unpropitious circumstances, no post-mortem examination was made.

The case is remarkable in its sudden and unexpected termination, which was truly appalling. True pains, which were never violent, occupied but little over an hour, cutting themselves off suddenly, as if conscious of utter incompetency. So far as known, the rupture may be said to have been, etiologically, idiopathic and almost spontaneous.

The foregoing history was recently (July 20) forwarded to Dr. James D. Trask, as appropriate to his forthcoming supplement to his valuable labors on this subject. I regret to say that his family have returned the same to me, as requested (after taking a copy), with the information that the doctor is absent, being compelled by ill health to cease work, and that the promised article is not ready. An opportunity is thus afforded for an additional observation.

It is well known that a more limited view of the causes of this accident than this case would seem to authorize, is maintained by Bandl and others. His conclusion, as drawn from thirty-two cases as observed by himself, and as taken from the records of the Vienna Lying-in Hospital, is as follows (I quote from the last No. of this journal, p. 289): "He has not found, in one single case, that pathological change in the substance of the uterus, which has so generally been assigned as a predisposing cause, more especially in multiparæ. The uterus was always thick, well contracted, high up, and the cervix very thin." . . . "The fissure was nearly always found in the cervix." . . . "He believes the rupture is always due to disproportion, and agrees with Chiari, Braun, and Spaeth, in considering that the abnormality is due to an excessive thinning of the cervix, occurring during labor." . . . "In normal circumstances the cervix is drawn over the head of the child by the muscular uterus; the *orificium internum* remaining about on the level of the brim of the pelvis. If there is a disproportion, which does not allow the presenting part to descend into the pelvis, the cervix is abnormally stretched," etc. "He believes that rupture can be recognized as threatening, when the internal orifice gradually ascends, whilst the cervix stretches, and the fundus acquires a lateral position."

The foregoing rationale is entirely unsatisfactory, as applied to the case before us. The "lateral," or anterior obliquity of the uterus existed at the outset, and was not the product of labor, and yet, doubtless, furnished a part, or the whole, of the "disproportion" claimed as necessary. It was not as excessive as sometimes noticed where the evil has been overcome without great difficulty, and yet its action alone was, probably, inadequate to the result.

As to the thinning of the cervix by the force of labor, in this case the stress, violence, and continuance of the latter were entirely inadequate to produce so grave a pathological condition. Very probably such thinning existed, but it must have originated prior to labor. Besides, we are often informed of cases where the rupture occurred in the "fundus," or "body" of the uterus, as shown by the autopsies.

As a practical appreciation of the foregoing, may we not deduce the principle, that "anterior obliquity" may constitute, in certain relations, a more serious obstacle to delivery than is usually taught, or believed?—*Am. Jour. of Med. Sciences.*

